# The Mother’s Autonomy in Decision Making (MADM) scale: Patient-led development and psychometric testing of a new instrument to evaluate experience of maternity care

**DOI:** 10.1371/journal.pone.0171804

**Published:** 2017-02-23

**Authors:** Saraswathi Vedam, Kathrin Stoll, Kelsey Martin, Nicholas Rubashkin, Sarah Partridge, Dana Thordarson, Ganga Jolicoeur, the Changing Childbirth in BC Steering Council

**Affiliations:** 1 Birth Place Research Lab, Division of Midwifery, University of British Columbia Vancouver, British Columbia, Canada; 2 School of Population and Public Health, University of British Columbia, Vancouver, British Columbia, Canada; 3 Department of Obstetrics and Gynecology, University of California San Francisco, San Francisco, California, United States of America; 4 Residency Program, Department of Family Practice, University of British Columbia, Vancouver, British Columbia, Canada; 5 Midwives Association of British Columbia, Vancouver, British Columbia, Canada; University of South Australia, AUSTRALIA

## Abstract

Shared decision making (SDM) is core to person-centered care and is associated with improved health outcomes. Despite this, there are no validated scales measuring women’s agency and ability to lead decision making during maternity care.

**Objective:**

To develop and validate a new instrument that assesses women’s autonomy and role in decision making during maternity care.

**Design:**

Through a community-based participatory research process, service users designed, content validated, and administered a cross-sectional quantitative survey, including 31 items on the experience of decision-making.

**Setting and participants:**

Pregnancy experiences (n = 2514) were reported by 1672 women who saw a single type of primary maternity care provider in British Columbia. They described care by a midwife, family physician or obstetrician during 1, 2 or 3 maternity care cycles. We conducted psychometric testing in three separate samples.

**Main outcome measures:**

We assessed reliability, item-to-total correlations, and the factor structure of the The Mothers’ Autonomy in Decision Making (MADM) scale. We report MADM scores by care provider type, length of prenatal appointments, preferences for role in decision-making, and satisfaction with experience of decision-making.

**Results:**

The MADM scale measures a single construct: autonomy in decision-making during maternity care. Cronbach alphas for the scale exceeded 0.90 for all samples and all provider groups. All item-to-total correlations were replicable across three samples and exceeded 0.7. Eigenvalue and scree plots exhibited a clear 90-degree angle, and factor analysis generated a one factor scale. MADM median scores were highest among women who were cared for by midwives, and 10 or more points lower for those who saw physicians. Increased time for prenatal appointments was associated with higher scale scores, and there were significant differences between providers with respect to average time spent in prenatal appointments. Midwifery care was associated with higher MADM scores, even during short prenatal appointments (<15 minutes). Among women who preferred to lead decisions around their care (90.8%), and who were dissatisfied with their experience of decision making, MADM scores were very low (median 14). Women with physician carers were consistently more likely to report dissatisfaction with their involvement in decision making.

**Discussion:**

The Mothers Autonomy in Decision Making (MADM) scale is a reliable instrument for assessment of the experience of decision making during maternity care. This new scale was developed and content validated by community members representing various populations of childbearing women in BC including women from vulnerable populations. MADM measures women’s ability to lead decision making, whether they are given enough time to consider their options, and whether their choices are respected. Women who experienced midwifery care reported greater autonomy than women under physician care, when engaging in decision-making around maternity care options. Differences in models of care, professional education, regulatory standards, and compensation for prenatal visits between midwives and physicians likely affect the time available for these discussions and prioritization of a shared decision making process.

**Conclusion:**

The MADM scale reflects person-driven priorities, and reliably assesses interactions with maternity providers related to a person’s ability to lead decision-making over the course of maternity care.

## Introduction

A move towards more person-centered care lies at the heart of global health care reform [1,2]. Indicators of person-centered care (including involvement in decision making) for acute care hospital patients are now collected on a national level in Canada [3]. A systematic review of 137 articles concluded that involvement in decision making, the quality of the provider-patient relationship, and the amount of support received from care providers are three main factors that influence women’s satisfaction with their birth experiences [4]. Shared decision making is considered a cornerstone of person-centered care and is associated with improved health outcomes [1,5–8], even when patients prefer not to participate in decision making. A more recent systematic review of 39 studies that examined the association between shared decision making and outcomes found that 54% of affective-cognitive outcomes (e.g. satisfaction with care), 37% of behavioural (e.g. diet, exercise, stress management) and 25% of health outcomes (e.g. symptom improvement, general health ratings) were positively and significantly associated with shared decision making. Decisional conflict was associated with shared decision making (SDM) in one of the studies [9].

Person-led decision making is especially important during pregnancy and birth, yet the inability to participate in decision making is commonly reported by childbearing women in English speaking countries [10]. In a qualitative study, Baker et al [11] reported that women felt that they were treated like children, were intimidated by care providers, had little control over the decision-making process, did not receive enough information about different options, and received interventions that were contrary to their preferences. Women had a desire to be better informed about why certain procedures were necessary and what the outcome might be. The authors concluded that this inadequate information provision and feeling of loss of control can be remedied by improved communication between care providers and childbearing women.

As a result, international health agencies emphasize the importance of respectful dialogue and service-user involvement in decision making during maternity care [12,13]. While several instruments have been developed to assess SDM in health care [9], or the quality of prenatal care received [14], none have focused on informed choice discussions between providers and during maternity care. Moreover, to our knowledge, none of the validated SDM scales were designed by service users. Caron-Flinterman noted that patients’ experiential knowledge “contributes to the relevance and quality of biomedical research” [15].

In the Changing Childbirth in BC research project, a steering group of women of childbearing age from different cultural and socio-economic backgrounds engaged multiple stakeholders, as well as leaders from NGOs, researchers, and community agencies, to examine women’s experiences with maternity care in British Columbia (BC). Together they designed a mixed-methods study to explore topics that have not previously been detailed—women’s preferences for model of care; perceptions of reasons for intervention; access to midwifery care; and experiences of autonomy, respect, discrimination, or coercion, when participating in a shared decision making process. This paper describes the resulting development and testing of a new scale to assess women’s autonomy and role in decision making throughout the course of pregnancy.

## Methods

The Vancouver Foundation funded our provincial, community-led participatory action project, and the research support necessary for development of this scale. The Steering Committee was composed of community members and leaders from Immigrant Services Society of British Columbia, Women in2 Healing, Midwives Association of British Columbia, Access Midwifery, Strathcona Midwifery Collective, clinicians and maternity care researchers from the Department of Family Practice at UBC, Children and Women’s Hospital, and the Women’s Health Research Institute. The core Steering Committee comprised 10 people at first, and expanded to 18 to include more women representing vulnerable populations.

The research team self-organized into four different work groups, adding some additional community members to each to ensure representation of the perspectives of the four sub-populations: women marginalized by economic and social barriers to health, formerly incarcerated women, immigrant and refugee populations, and all other maternity service users.

The team used existing public e-lists and member databases to canvas the community about areas for study, and preferred modes of data collection. Through community consultations with 1300 women, the work groups agreed on key topics for study, a mixed-methods approach, recruitment strategies, and a timeline for data collection.

### Survey development

Following a broad literature review on their chosen topics, the team adapted or modified previously validated items, and generated new items to populate four versions of a cross-sectional online survey and focus group questions. After an extensive content validation process, including expert panel review by all Steering Committee members and all work group members, the final instrument included 130 core items that collected information on demographics, access to maternity care, preferences for model of care, maternal and newborn outcomes, knowledge of midwifery care, and experience of care including the process of decision-making.

Given the length of the survey we reduced the burden to participants by using an online platform with logic branching to ensure that participants only answered questions that related to their experience; by allowing skipping of any question, except the eligibility criteria; by making it optional for women with previous childbirth experiences to report on care during 1, 2 or 3 maternity care cycles; and by setting the survey save functions to allow respondents to complete the survey over more than one session.

The survey was edited for lay language (i.e. a grade 8 reading level), and then pilot tested with several women from the target populations. Final revisions were made, based on the feedback of women who pilot tested the survey. For instance, issues with survey logic branching were corrected at this stage and some items were reworded, to improve clarity.

#### Measuring experiences of decision making in maternity care

To construct the decision-making section of the survey, the community members reviewed several previously validated instruments and found that, while there were other tools surrounding shared decision making, there were no scales that measured the ability of the person to lead the decision making, or the degree to which their preferences were respected. Moreover, all of the published tools included a broad range on indicators of quality in prenatal care or were developed for general medical treatment options (e.g. for cancer or diabetes), and thus not always relevant to maternity care where decisions impact both mother and baby. Our community team members wanted a measure that did not pathologize pregnancy but could assess involvement in all types of decision making during the entire course of pregnancy. They also noted that none of the existing instruments measured the impact of time allowed for decision making. Hence, to respond to the stated community concerns, we adapted the language of previously validated tools and generated new items.

Of a total 31 items describing preferences for and experiences of decision making over the childbearing cycle, 14 items addressed the nature of communication with providers, and seven items specifically measured women’s perception of their role and agency when participating in a shared decision making process (responses on a six-point Likert scale, range of scores 7–42). Higher scores indicate that women had greater agency and autonomy when engaging in an SDM process with a maternity care provider. Four of these items were adapted to the maternity care context from the previously validated 9-item Shared Decision Making Questionnaire [16] that had been administered to 2351 German primary medical care patients to describe their consultations about a specific health problem, illness or complaint. For example, the team changed ‘My doctor told me there are different options for treating my medical condition’ to ‘My (*family physician/obstetrician/midwife*) told me that there are different options for my maternity care’.

The research team then designed three new items, reflecting the priorities identified by ongoing community consultation, to assess the ability of women to lead decision-making:

- I was given enough time to thoroughly consider the different care options
- I was able to choose what I considered to be the best care options
- My (*family physician/obstetrician/midwife*) respected that choice

Finally, most other instruments measure interactions between patients and a single type of provider, usually physicians. In contrast, respondents to the Changing Childbirth in BC survey could indicate that their responses referred to any of five types of maternity professionals (midwife, family physician, health centre nurse, obstetrician, or other) when they described their experience. See Table 1 for a full list of scale items.

**Table 1:** Scale items—Mothers Autonomy in Decision Making (MADM)^1^.

Please describe your experiences when making decisions and choosing options for care during this pregnancy. (*Auto-populated with provider type*)
My _______ asked me how involved in decision making I wanted to be
My _____ told me that there are different options for my maternity care
My ______ explained the advantages and disadvantages of the maternity care options
My ________ helped me understand all the information
I was given enough time to thoroughly consider the different maternity care options
I was able to choose what I considered to be the best care options
My _________ respected that choice

^1^.Response options are (1) Completely disagree; (2) Strongly disagree; (3) Somewhat disagree; (4) Somewhat agree; (5) Strongly agree; (6) Completely agree

To assess the scale items within the context of their preferences for decision making, we asked women how important it is for them to lead the decisions about their pregnancy, birth and baby care. Response options included ‘very important’, ‘important’, ‘somewhat important’ and ‘not important’. We also asked participants to rate whether they were satisfied or dissatisfied with their ability to participate in decision making in different periods over the course of care: 1) pregnancy, 2) labour and birth, 3) after the birth, 4) baby care or 5) none of the above.

### Data collection

Women of childbearing age across BC were recruited via email, community list-serves, NGO websites and social media outlets (i.e. convenience sampling). The survey was accessible online, and for women with barriers to this format, also via assisted data entry by a trained volunteer at the site of their care. Recruitment posters and postcards were printed and disseminated widely in community centers, grocery stores, and maternity shops; in midwife and family physician offices; and at BC Women and Children’s Hospital, the largest referral maternity hospital in the province. To introduce the study to maternity care providers and to encourage recruitment, the first author also made presentations at a UBC Department of Family Practice meeting, and hospital department meetings in Richmond, Vancouver and Victoria, and at the provincial Annual General Meetings of the College of BC Midwives. Data was collected between January and June 2014.

The University of British Columbia (UBC) provided ethics approval for the Changing Childbirth in BC Study. Participants who clicked on the survey link were first taken to an informed consent page that explained the purpose of the study, and described the study team, potential impacts and consent procedures. Potential participants were informed that their participation in the study was entirely voluntary, that they could skip items, and that they could decide to leave the study at any time. Participants were further informed that by continuing on to fill out this survey, they consented to participate in the study. This consent procedure is standard for online surveys that are administered in British Columbia and was approved by the University Ethics Board.

### Sample

Women could report on their experiences during two previous pregnancies and the current pregnancy (if applicable). If women had more than one care provider during a single pregnancy, they had the option of describing their experiences with up to three different care providers. For the purposes of the current analysis, we excluded responses about childbirth experiences from outside of British Columbia and about health center nurses and “other” care providers from the dataset, to focus on the three types of primary maternity care providers available in BC (i.e. midwives, family physicians, or obstetricians). This resulted in a sample of 2051 women reporting on 3400 care provider experiences.

Of women who responded to the scale items on the survey (n = 2051), the majority (88.6%) experienced their last pregnancy within 5 years of data collection. Only 47 women experienced their last pregnancy more than 10 years ago. By linking postal codes to provincial data by health authority, we determined that our sample was closely matched to the geographic distribution, and socioeconomic and age profile of childbearing women in BC. With respect to visible minorities our sample was under-represented. Women in the sample also reported higher education on average than the general population of Canada. Reported family incomes in our sample resembled incomes of families in British Columbia in 2014.

To avoid multiple observations from the same woman, we excluded 886 care provider experiences reported by 379 women who had multiple providers during a pregnancy. Sample characteristics are reported for the 1672 women who saw a single care provider during pregnancy. Socio-demographic data pertain to the time of data collection, not the time of the pregnancy experience (with the exception of the women who were pregnant at the time of data collection). To assess the psychometric properties of the scale items, we analyzed responses from two groups: care provider experiences during the course of 1 or 2 previous pregnancies (n = 2271), and experiences of women who were pregnant at the time of data collection (n = 243), for a total of 2514 care provider experiences. We report results separately for each pregnancy, to demonstrate that findings are replicable.

### Data analysis

We estimated internal consistency reliability with Cronbach’s alpha. To examine how well each item contributes to the overall measurement of women’s role in decision making, we generated corrected item- to-total correlations. High correlation coefficients represent ‘good items’ that should be included in the scale. In addition, when all corrected item- to- total correlations exceed 0.45 there is strong evidence of the uni-dimensionality of a scale [17]. We performed unweighted least squares factor analysis (no rotation), to examine the factor structure of the scales and to determine the construct validity of the new measure.

We created scale scores (i.e. the sum of the items) for women who responded to all seven scale items, i.e. women who missed any items or marked one or more items as ‘not applicable’ were not included. We report median scores because scale scores were not normally distributed. Median scores are reported for the full sample, and separately for women who saw midwives, family doctors and obstetricians. We calculated descriptive statistics for women who reported their preferences for and satisfaction with role in decision-making. The relationship between care provider type and dissatisfaction with involvement in decision making was assessed, using the Chi-square test. Finally, we examined the average length of prenatal appointments (< 15 minutes, 15–30 minutes, 31–60 minutes and > 60 minutes), in association with autonomy scores and stratified by care provider type.

## Results

Of the 2514 care provider experiences reported, 68.5% (n = 1723) related to midwifery care, 19.9% (n = 500) to care provided by family physicians, and 11.6% (n = 291) to obstetric care; 9.7% (n = 243) care provider experiences were submitted by women who were pregnant at the time of data collection.

The average age of women at the time of data collection was 32.6 years; 4.5% self-identified as vulnerable (i.e. women who arrived as immigrants or refugees in Canada within the last 5 years and/or women with a history of substance use, poverty, homelessness or incarceration). The majority of women identified as White (92.5%), 1.6% identified as Chinese, and 1.8% as First Nations, Inuit, or Métis. The remainder reported other ethnicities. Of the women surveyed, 8.2% reported family incomes < 30,000 and 37.4% reported incomes exceeding $91,000. While most women had completed college or university, 10.1% reported high school as the highest level of education completed.

Eighteen women (1.1%) were expecting twins; 10.2% of women reported one or more medical or social risk factor during pregnancy (high blood pressure, diabetes, problems with baby’s growth, problems with baby’s health, depression, lack of social support during pregnancy, or housing difficulties).

The majority of women (90.8%) said it was *very important* or *important* that they lead decisions about their care. When asked whether they were satisfied with their ability to participate in decision making during pregnancy, labour and birth, and/or postpartum (including baby care), 6.2% of women were dissatisfied during pregnancy, 15.2% during labour and birth, 15.8% after the birth, 12.9% with baby care and 2.7% were not satisfied at any point during pregnancy. Women with physician carers were consistently more likely to report dissatisfaction with their involvement in decision making (see Table 2).

**Table 2:** Dissatisfaction with decision-making experience, by care provider type.

Women dissatisfied with experience of decision-making:	Family Physician	Obstetrician	Midwife	p
During pregnancy	47 (16.4)	35 (19.7)	21 (1.7)	< 0.001
During labour/birth	77 (29.4)	52 (32.3)	88 (8.8)	< 0.001
After birth	68 (26.0)	65 (40.4)	92 (9.2)	< 0.001
About newborn care	47 (17.9)	56 (34.8)	80 (8.0)	< 0.001
At any time	18 (6.9)	10 (6.2)	10 (1.0)	< 0.001

### Reliability and validity of MADM

**Fig 1:**
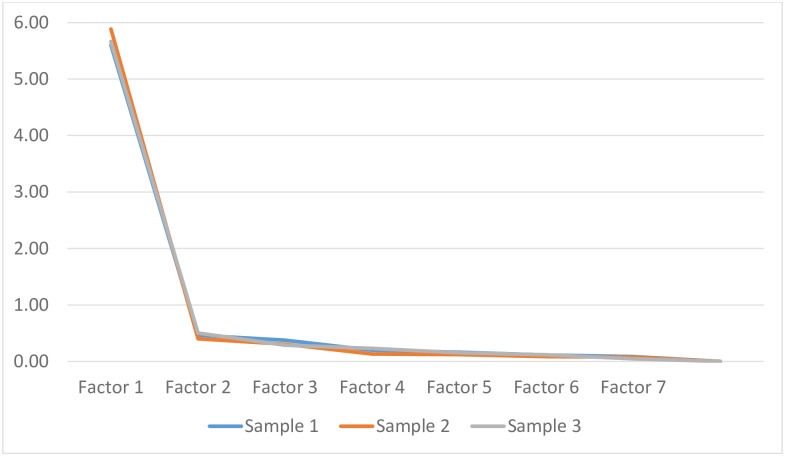
Scree plots for Samples 1, 2, and 3. When examining construct validity indicators separately for physician and midwifery consumers in Sample 1, we found that factor loadings for women who were cared for by family physicians ranged from 0.73–0.88 (n = 264), 0.80–0.92 for women under the care of obstetricians (n = 150) and 0.64–0.91 for midwives (n = 927). For all care provider groups the scree plots showed one factor with an Eigenvalue above 5; all other Eigenvalues fell clearly below 1 (see Fig 2).

Cronbach alphas, for the seven items that measured autonomy and role in decision making, exceeded 0.90 in each subsample (see Table 3). All corrected item-to-total correlations for the first pregnancy exceeded 0.7 and most exceeded 0.8. These findings could be replicated with the second sample (i.e. women reporting experiences during a different pregnancy) and third sample (women who were pregnant at the time of data collection) (see Table 4). Because all seven items were highly correlated with the sum of all other items, we concluded that they formed a uni-dimensional scale, measuring autonomy in decision making. An examination of Eigenvalues, factor loadings and the scree plot of MADM items further supported the uni-dimensionality and construct validity of the scale. For each sample, one Eigenvalue was larger than 1, scree plots exhibited a clear 90 degree angle (see Fig 1), and the factor analysis generated one factor, with loadings ranging from 0.74–0.93 for sample 1, 0.76–0.95 for sample 2 and 0.79–0.93 for sample 3. To honor the participatory construction of the instrument, we named the scale Mothers’ Autonomy in Decision Making (MADM).

**Table 3:** Cronbach alphas for MADM scale, full sample and by care provider type.

	Pregnancy 1	Pregnancy 2	Currently pregnant
MADM- All	0.96	0.97	0.96
MADM- MW	0.93	0.96	0.96
MADM-FP	0.95	0.95	--
MADM-OB	0.95	0.97	--

MW: midwife; FP: family physician; OB: obstetrician

--Alphas for sample sizes < 20 are not reported

**Table 4:** Corrected item to total correlations and factor loadings of MADM items.

Scale item		Corrected ITTC	Factor loadings
My ________ asked me how involved in decision making I wanted to be	P1	0.73	0.74
	P2	0.75	0.76
	CP	0.78	0.79
My ______told me that there are different options for my maternity care	P1	0.86	0.88
	P2	0.91	0.93
	CP	0.85	0.87
My ______explained the advantages/disadvantages of the maternity care options	P1	0.86	0.88
	P2	0.90	0.91
	CP	0.88	0.90
My_________ helped me understand all the information	P1	0.90	0.92
	P2	0.93	0.95
	CP	0.91	0.93
I was given enough time to thoroughly consider the different care options	P1	0.90	0.93
	P2	0.93	0.95
	CP	0.87	0.90
I was able to choose what I considered to be the best care options	P1	0.88	0.91
	P2	0.91	0.93
	CP	0.87	0.91
My _______ respected that choice	P1	0.84	0.87
	P2	0.87	0.89
	CP	0.85	0.89

Pregnancy 1 –P1; Pregnancy 2 –P2; Currently Pregnant—CP; ITTC- Item to total correlations

**Fig 2:**
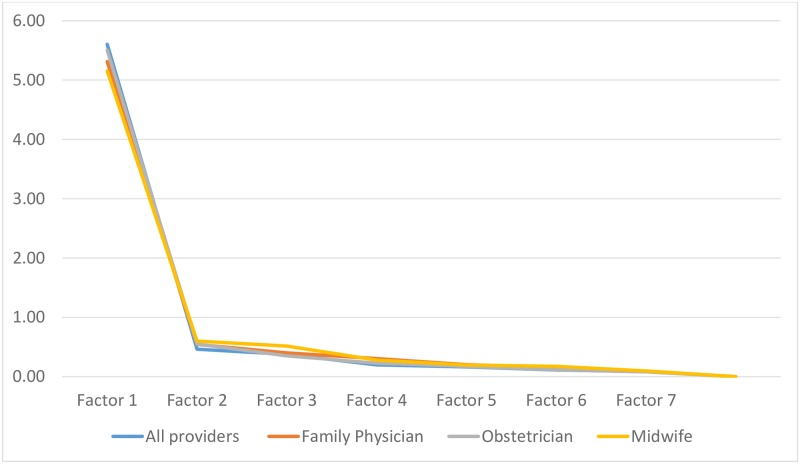
Scree plots by provider type (Sample 1).

### Median scale scores

**Fig 3:**
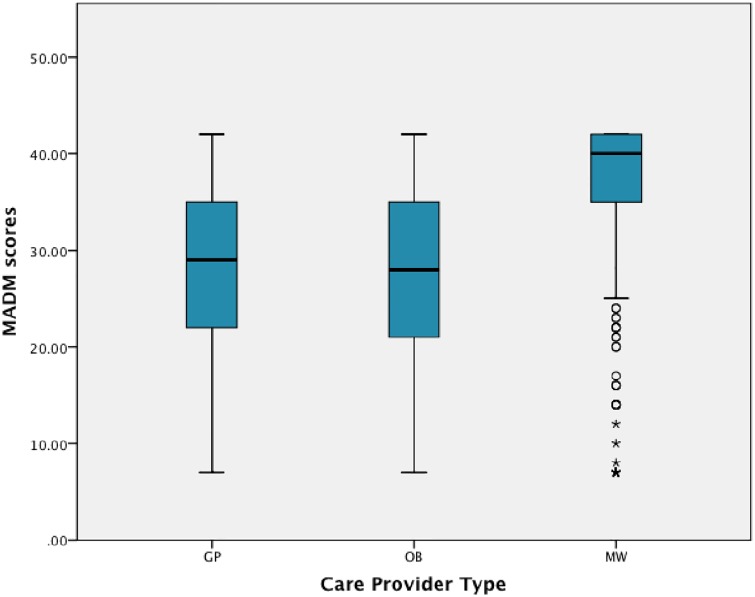
Box plot: MADM scores by care provider type (Sample 1 = 1344). Median and interquartile range of MADM scores by care provider group for pregnancy 1 (n = 1344). The horizontal line inside each box represents the median score for each provider group, and the upper and lower boundaries of each box represent the upper and lower quartiles. The vertical lines represent the range of scores, excluding outliers, which are represented by open circles and asterisks.

MADM median scores were highest for midwifery clients (40.0 in pregnancy 1 and 41.0 in pregnancy 2 and among pregnant women), and 10 or more points lower for women who saw physicians during pregnancy (see Table 5). See Fig 3 for a graphic representation (box plots) of scale scores by provider type.

**Table 5:** MADM median scale scores, full sample and stratified by care provider.

	Pregnancy 1		Pregnancy 2		Currently Pregnant	
	n	Median	n	Median	n	Median
MADM-all	1344	38.0	571	40.0	190	40.5
MADM-MW	927	40.0	433	41.0	162	41.0
MADM-FP	266	29.0	93	30.0	17	--
MADM-OB	151	28.0	45	31.0	11	--

MW: midwife; FP: family physician; OB: obstetrician

--Medians for sizes < 20 are not reported

Note: Some responses were excluded from this analysis because women did not complete all MADM scale items or checked ‘not applicable’ on one or more items: 252/1596 (15.8%) for sample 1, 104/675 (15.4%) for sample 2 and 53/243 (21.8%) for the women who were pregnant at the time of data collection

### Time and experience of autonomy

**Fig 4:**
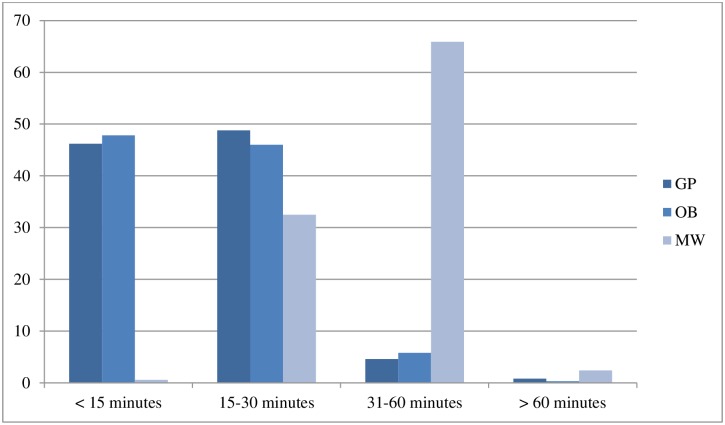
Average prenatal appointment lengths, by care provider type (n = 1723).

Increased time for prenatal appointments was associated with higher scale scores (see Table 6), and there were significant differences between providers with respect to average time spent in prenatal appointments (see Fig 4).

**Table 6:** MADM median scale scores, by average length of prenatal appointments.

	Pregnancy 1		Pregnancy 2		Currently Pregnant	
	n	Median	n	Median	n	Median
< 15 minutes	191	23.0	73	25.0	9	--
16–30 minutes	501	36.0	207	39.0	74	39.5
31–60 minutes	649	41.0	292	42.0	106	41.5
>60 minutes	25	41.0	5	--	11	--

-- Medians for sample sizes < 20 are not reported

Midwifery care was associated with higher MADM scores, compared to physicians, even during short prenatal appointment (less than 15 minutes). For sample Pregnancy 1, women who saw midwives with appointment times of < 15 minutes had a median MADM score of 30, compared to a median MADM score of 23 among those who saw obstetricians, and 22 among women who saw family physicians during short appointments. We obtained similar findings for women who reported prenatal appointments that lasted between 15–30 minutes: those with midwives had median MADM scores of 39, and those cared for by OBs scored 34.5 and those with family physicians scored 33. Among the women who said it was ‘*very important’ or ‘important’* to lead the decisions around their care (90.8%), and who were dissatisfied with their experience of decision making at any time during the maternity care cycle, MADM scores were very low (median 14).

## Discussion

We introduced a new scale to measure women’s experiences with autonomy in decision making. The Mothers Autonomy in Decision Making (MADM) scale is a reliable instrument for assessment of experiences among women who are reflecting on past childbearing experiences as well as women who are currently pregnant. It is uni-dimensional and internally consistent. This new scale was developed and content validated by community members representing various populations of childbearing women in BC. Notably, it uniquely allows assessment of women’s ability to *lead* decision making, whether they have enough time to consider their options, and whether providers respect women’s choices.

When women are encouraged by clinicians to have a key role in decision making, several benefits have been observed: increased satisfaction with the consultation process, reduction in options/procedures that are over-used, and increased sustainability of health systems [18]. However, a review of barriers and facilitators of shared decision-making among care providers revealed three salient factors: care provider motivation, the belief that outcomes are enhanced with shared decision making, and the perception that shared decision making has a positive impact on the clinical process [19]. Our study elicits some differences between types of providers with respect to the level of autonomy and involvement that women experienced.

The importance of pregnant women’s autonomy and the aspiration to shared-decision making have been affirmed by statements from medical professional associations [20], including, the Institute of Medicine [21], the American College of Obstetricians and Gynecologists [22] the National Institute for Clinical Excellence [13], and the Society for Maternal Fetal Medicine [20]. However, contentious debates continue among professionals over the meaning of and limits to autonomy around issues like elective cesarean sections, genetic testing, and place of birth [23, 24]. More in-depth, obstetrician-driven explorations of the principles of autonomy, beneficence, and respectful care are emerging [25]. They are an important addition to the current approach to evidence-based obstetrics that has been criticized for an emphasis on risk measurement to the detriment of women’s autonomy [23,25,26]. However, despite these efforts in the fields of medical ethics and anthropology, quantitative measures of women’s autonomy are sorely lacking. Measures such as the MADM scale could be integrated into clinical trials to explore associations between obstetric interventions and autonomy, potentially implemented as a national quality measure, or even employed as a provider performance indicator—taking autonomy out of the theoretical realm of professional statements and placing it as a standard of practice.

In our sample, women who experienced midwifery care reported increased agency and autonomy in decision making, compared to women under physician care. Midwives have been recognized for prioritizing the importance of shared decision making, and women centered care [27,28]. However, shared decision-making requires taking the time and willingness to engage in evidence-based discussions with women about the pros and cons of different care options. Other instruments that assess the quality of prenatal care incorporate this important dimension, i.e. whether people have sufficient time to discuss their options and make decisions [14]. Current differences in models of care, health professional education, regulatory standards, and compensation for prenatal visits likely affect the time available for these discussions and the emphasis placed on the shared decision making process.

### A patient-driven health service priority

Our study results demonstrate that physicians are spending less time than midwives during prenatal visits, and that reduced time during prenatal appointments is associated with lower autonomy scores. These findings are especially significant in BC [29] where over the last decade, the birth rate is rising, there has been a reduction in the number of family physicians and obstetricians who provide maternity care, and midwives have been added to the register. Rural and remote areas are particularly affected, and many pregnant women travel long distances to urban centers to access care [30]. In addition, family physicians’ mandate is to provide universal access to care for all types of people, from cradle to grave, despite a chronic physician shortage. Similarly, obstetricians-gynecologists must provide consultation and care for all women with complex gynecologic conditions and at-risk pregnancies, including referrals from family physicians and midwives. The need to ensure universal access to care in this provincial context may have fostered an environment where detailed discussions about options and preferences are sometimes deferred, or are a lower priority, because of time constraints.

Also, since the bulk of people that family physicians and obstetricians see in a session have some pathological medical condition, the physician’s necessary emphasis on addressing medical comorbities may leave little time to explore normal physiologic processes. In current medical practice, detailed discussions with medical patients about options and preferences are focused on conditions that require complex care planning (eg. chemotherapy, palliative care) [9]. In contrast, a “risk” focus for informed consent conversations has been attributed to modern obstetric care [31,32]. Unfortunately, discussions that center on evidence-based options to address potential pathology may inadvertently create a perceived “expert knowledge” imbalance between caregiver and the pregnant person, further undermining the person’s sense of autonomy.

Because of widespread community-based advocacy for inclusion of midwifery within the BC maternity care system, midwifery care was introduced in 1998. Midwifery is now the fastest growing primary maternity profession in BC. Midwives in BC offer primary care to healthy pregnant women and their normal newborn babies from early pregnancy, through labour and birth, and up to three months postpartum. Following extensive community consultations in the 1990s, BC established a provincial midwifery model of practice which includes regulatory requirements that midwives provide, and demonstrate that they offer: 1) continuity of carer; 2) informed decision making; 3) women-centered care; and 4) choice of birthplace.

In our sample, midwives typically spent 30–60 minutes with women in each prenatal visit, whereas almost half of physicians spent less than 15 minutes. Women’s sense of autonomy increased with more time for prenatal appointments. Notably, in BC the payor model for midwives acknowledges the additional time needed to establish relationship-based continuity of care and to engage in an informed decision making process. To ensure that caseloads are reasonable enough to both provide a living wage, and to ensure adequate face-to-face time per person, midwives are paid a single comprehensive fee for each course of care and are capped on the number of persons they can be compensated for annually. Family physicians and obstetricians, in contrast, are paid in a fee-for-service model that incentivizes higher caseloads and procedures, without addressing impacts on quality of relationships or person-led care.

At the same time, care providers vary in their attitudes towards medical management of birth and their comfort with letting go of control [24,33]. Some providers express the view that being in control is part of their job and a way to avoid medico-legal issues. Other providers value women’s agency and shared decision making. Some care providers recounted how the evidence can easily be presented in a way that will maximize compliance with care providers’ preferred course of action. Other care providers believe that women like to defer to medical authority even after their care provider has engaged them in an informed choice discussion [33].

However, the majority of respondents in our study, regardless of type of care provider, indicated a preference for leading decision making. Women who were dissatisfied with their role in decision making during pregnancy, birth, and postpartum had very low MADM scores. These findings draw attention to the importance of asking women how important it is for them to lead decision around their care and under which circumstances they want to share decision making or defer to the recommendations of their care provider(s). Previous research with health care consumers in the UK found that a desire to be involved in decision making is context specific. Consumers felt that health professionals ‘should seek involvement to the level that the consumer desires’. Consumers desired less involvement in emergency situations, because they expected health professionals to direct care, to the best of their professional expert knowledge [34].

Finally, medical students and residents must navigate these attitudes and preferences as they formulate their own approach to informed consent. Within the educational context, the value of deferring to women as “experts” about their own physiologic responses is not consistently emphasized. Care provider education could prepare both medical and midwifery trainees for the realities of person-centered practice. The scale is brief and easy to administer, making it ideal to capture the patient perspective on the performance of health professional learners on clinical education encounters.

### Strengths and limitations

Our scale development through a community-led participatory approach makes the MADM scale particularly relevant to person-centered care. The psychometric testing of the scale in a large geographically distributed provincial sample, with replication of results in two further samples, supports the reliability and construct validity of the scale. Internal consistency reliability of MADM was excellent for the full sample with a mix of care providers, and also across specific provider groups.

The convenience sampling frame (including recruitment via social media) is a limitation of this study, because it prevents us from calculating a response rate as it is unknown how many eligible participants were invited to the study/saw the advertisement. Furthermore, the accuracy of results might be impaired by recall bias. The chance of recall bias in this study is minimized because the majority of women who responded to the survey experienced their last pregnancy within 5 years of data collection (88.6%). Research suggests that women’s recall of their birth experiences, even if asked 10–15 years after the event is very accurate, when compared to medical charts [35,36].

Finally, we note that while we had 4.5% participation from the target vulnerable groups, and 8.2% of women reported low family income (<30K), results may not be representative of the experiences of vulnerable populations in BC. Nonetheless, we used a participatory approach to design all survey items and to recruit survey respondents. One of the four work groups represented maternity care recipients in the province that did not self-identify as from a vulnerable group, but all four work groups agreed on a core set of items to be assessed in all populations, so that we could compare findings across groups. We partnered with NGOs serving vulnerable populations and paid participants an honorarium, offered food, chose convenient locations and had childcare on site, to reduce barriers to research participation. In addition, anticipating common barriers to participation from the 3 vulnerable populations, our primary method of data collection for those populations was via focus groups on the same 4 topic areas. Preliminary results of the qualitative data indicate that the themes strongly support our quantitative findings and the triangulated results will be reported elsewhere.

## Conclusion

We have created a reliable and valid scale that can evaluate the process of decision making in maternity care. The Mothers Autonomy in Decision Making (MADM) scale will be especially valuable in a field that has a scarcity of reliable tools to evaluate patient experience. The adaptation of previously validated items and generation of new items by community members strengthens the relevance of the scale to person-centered care.

In an era of increased demand and value for patient involvement and self-determination in health care, an instrument that allows women to quantify their ability to participate in decision making can inform quality assurance and improvement of health services and health professional education. Health care systems that prioritize person-led care may benefit from using the MADM scale to assess the agency accorded to service users when making decisions in different models of maternity care. Global applications may assist health policy makers to appraise evidence of respectful maternity care.
